# Nutrient acquisition of an underground and mycoheterotrophic orchid

**DOI:** 10.1093/aobpla/plag027

**Published:** 2026-06-02

**Authors:** Denisele Neuza Aline Flores-Borges, Laís Soêmis Sisti, Cristina Antunes, Mauro Brum, Cristina Máguas, Juliana Lischka Sampaio Mayer

**Affiliations:** Department of Plant Biology, Institute of Biology, University of Campinas—UNICAMP, P.O. Box 6109, Campinas, SP 13083-970, Brazil; Faculty of Sciences, Center for Ecology, Evolution and Environmental Changes, University of Lisbon, Campo Grande 016, Lisbon 1749-016, Portugal; Department of Plant Biology, Institute of Biology, University of Campinas—UNICAMP, P.O. Box 6109, Campinas, SP 13083-970, Brazil; Faculty of Sciences, Center for Ecology, Evolution and Environmental Changes, University of Lisbon, Campo Grande 016, Lisbon 1749-016, Portugal; Graduate Program in Ecology (PPGECO), Institute of Biological Sciences, Federal University of Pará, 01 Augusto Corrêa Street, Guamá University Campus, Belém, PA 66075-110, Brazil; Faculty of Sciences, Center for Ecology, Evolution and Environmental Changes, University of Lisbon, Campo Grande 016, Lisbon 1749-016, Portugal; Department of Plant Biology, Institute of Biology, University of Campinas—UNICAMP, P.O. Box 6109, Campinas, SP 13083-970, Brazil

**Keywords:** *Pogoniopsis*, Orchidaceae, mycoheterotrophy, achlorophyllous plant, fungal dependence, ectomycorrhizal fungi, saprotrophic fungi, stable isotope, starch, orchid root anatomy, tolypophagic, ptyophagic

## Abstract

Most species of orchids become autotrophic after the formation of the first leaf develops; however, some mycoheterotrophic species remain achlorophyllous at maturity and non-photosynthetic throughout their entire life cycles, depending on fungal associations for survival. However, the mechanisms governing carbon and nitrogen exchange between mycoheterotrophic plants and their associated fungi remain largely unexplored in neotropical regions. Studies with mycoheterotrophic orchids from tropical regions and different subfamilies contribute to a better understanding of the nutrition mode of these plants, and allow evaluation of the role that morphological and anatomical characteristics play in the mycoheterotrophic way of life. Samples of different individuals of *Pogoniopsis schenckii* were fixed and subjected to standard anatomical techniques for light microscopy, histochemical tests, transmission and scanning electron microscopy and stable isotope analysis. *Pogoniopsis schenckii* exhibits two distinct root types: one with acute apex and another with a rounded apex. Fungal hyphae were found in all roots but were not organized into typical pelotons; instead, they were distributed throughout the epidermis and cortex, including within cells associated with starch storage. Our isotopic results suggest that *P. schenckii* acquires carbon and nitrogen through its symbiotic fungi from the soil. The root system of *P. schenckii* exhibits two root morphotypes that differ in size, apex shape, and anatomy. The observed patterns of ^13^C and ^15^N abundances in *P. schenckii* suggest that this mycoheterotrophic orchid is associated with ectomycorrhizal fungi. Additionally, we described two trends in the degradation of the semi-coiled hyphae found within the epidermal and cortical cells of the two root morphotypes of this mycoheterotrophic species.

## Teaser

This study demonstrates that *Pogoniopsis schenckii*, a fully mycoheterotrophic orchid, relies on fungal partners as its primary source of carbon and nitrogen. By integrating stable isotope analysis with anatomical and morphological data, we show that distinct root morphotypes likely perform complementary functions related to nutrient uptake, storage, and fungal maintenance. These findings highlight the complexity and ecological flexibility of plant–fungus interactions in tropical forest ecosystems.

## Introduction

All species of the Orchidaceae family depend on the association with fungi to acquire nutrients during germination in natural environments, because their seeds are very small and rudimentary (Rasmussen and Rasmussen 2009). Most orchid species become autotrophic once their first leaves develop. However, certain mycoheterotrophic species remain achlorophyllous at maturity and are non-photosynthetic throughout their entire life cycle, depending exclusively on fungal associations for survival (Leake 1994, Merckx 2013). Mycoheterotrophic plants, which encompass over 200 orchid species (Leake 2004, Merckx 2013, Roy et al. 2013), constitute approximately 0.6% of the total species of the Orchidaceae family. Tropical forests provide dense canopies that create suitable habitat for plants that do not rely on light for nutrition. As a result, these ecosystems harbour a remarkable diversity of mycoheterotrophic species, including several rare taxa (Roy et al. 2009). Despite this, research on mycoheterotrophic plants has focused primarily on species from temperate regions (Taylor et al. 2002, Leake 2004, Roy et al. 2009, Bougoure et al. 2010).

Mycoheterotrophic species are typically more abundant in shaded habitats, such as the understory of tropical forests, where environmental conditions favour the presence of fungi—key components of biogeochemical cycles in these ecosystems (Merckx 2013). These fungi degrade organic macromolecules and actively mobilize nutrients, playing a central role in ecosystem nutrient cycling (Rasmussen and Rasmussen 2009). However, the mechanisms of carbon and nitrogen transfer between mycoheterotrophic plants and their fungal partners in neotropical regions are unknown. One of the primary factors limiting the geographic distribution of mycoheterotrophic plants is the availability of their fungal partners (Leake 1994). In temperate regions, these mycoheterotrophic plants generally exhibit two key characteristics: high fungal specificity and an indirect nutritional connection to nearby autotrophic plants through shared mycorrhizal networks, which mediate carbon and nitrogen transfer (Taylor et al. 2002, Leake 2004, Bougoure et al. 2010, Suetsugu et al. 2020, Zhang et al. 2024).

Carbon and nitrogen acquisition in mycoheterotrophic plants is strongly shaped by regional ecological conditions, particularly climate, soil nutrient dynamics, and the functional composition of fungal communities (Smith and Read 2008). In temperate ecosystems, pronounced seasonality, low primary productivity, and slow litter decomposition result in conservative nitrogen cycling. Under these conditions, mycoheterotrophic plants typically rely on stable, host-mediated carbon transfer through ectomycorrhizal networks, which link them indirectly to co-occurring photosynthetic plants (Bidartondo et al. 2002, Selosse and Roy 2009).

By contrast, neotropical forests are characterized by year-round primary productivity, rapid litter turnover, and intense nitrogen cycling, which enhance the availability of organic nitrogen pools and sustain high fungal functional diversity (Martinelli et al. 1999, Hättenschwiler et al. 2011). These conditions may confer greater nutritional flexibility in mycoheterotrophic plants, enabling associations with saprotrophic or endophytic fungi and reducing dependence on a single photosynthetic carbon source (Mayor et al. 2009, Merckx and Freudenstein 2010). Taken together, these contrasting ecological contexts provide a framework for understanding regional differences in carbon and nitrogen acquisition pathways in mycoheterotrophic plant–fungus interactions and form the basis for the hypotheses tested in this study.

Although the existence of a link between orchids and fungi is well known, <30% of the genera of the Orchidaceae family have had their endophytic fungal diversity investigated (Ma et al. 2015). The role of endophytic fungi may have been neglected, as mycorrhizal associations are considered crucial for orchid nutrition, particularly during early development phases (Bayman et al. 1997). Orchid mycorrhizal fungi form tangled hyphal structures known as pelotons (Leake 1994). Pelotons are typically described as densely coiled hyphal masses occupying the cortical cells of roots, though variations in their compactness and organization have been reported, with some species exhibiting loosely or semi-coiled pelotons depending on developmental stage or environmental context (Huynh 2004, Zhu et al. 2009, Sathiyadash et al. 2012). Early microscopy studies showed that pelotons are usually degraded within the orchid cells (Selosse et al. 2011, Kuga et al. 2014), a process long considered the primary mechanism of nutrient transfer from the fungi to orchid’s host cells (Burges 1939, Burgeff 1959). While this hypothesis was the most accepted, alternative views suggested that nutrient transfer could occur while pelotons were still alive (Mollison 1943, Hadley and Williamson 1971) has also been raised in ultrastructural analyses (Hadley et al. 1971).

The fungi are capable of colonizing both roots and rhizomes, developing within the parenchymal cells (Peterson et al. 2004, Rasmussen and Rasmussen 2009). Fungal partners of mycoheterotrophic plants often envelop the roots entirely to supply nutrients from the soil (Leake 2004, Cameron et al. 2006). The ecological relationship between fungi and mycoheterotrophic orchids is intriguing because the fungi can survive independently from plants, whereas orchids cannot survive, suggesting a largely unilateral relationship in which only the plant benefits (Rasmussen and Rasmussen 2009). Since fungi receive carbon from multiple photosynthetic species, unveiling the connections within this complex network of plants and fungi remains challenging. Moreover, the small microscopic size of many fungal structures makes *in situ* observations of carbon transfer difficult (Högberg 1997).

The natural abundance of stable nitrogen and carbon isotopes (δ^13^C and δ^15^N) provides a powerful tool for clarify the nutritional sources of mycoheterotrophic plants (Dawson et al. 2002, Post 2002, Gebauer and Meyer 2003, Roy et al. 2009, Liebel et al. 2010, Gomes et al. 2020). This approach has been widely applied to trace the origin of nitrogen and carbon acquired by mycoheterotrophic plants (Gomes et al. 2020). Previous studies demonstrate that mycoheterotrophic plants become significantly enriched in δ^13^C and δ^15^N compared to photosynthetic plants from the same location (Gebauer and Meyer 2003, Zimmer et al. 2008, Martos et al. 2009, Lee et al. 2015, Hynson et al. 2016, Ogura-Tsujita et al. 2018, Gomes et al. 2020). Such enrichment arises because fungi themselves exhibit enriched δ^13^C and δ^15^N values, with δ^15^N accumulating along trophic levels (Gebauer and Dietrich 1993, Gleixner et al. 1993, Högberg 1997, Liebel et al. 2010). As mycoheterotrophic plants depend on fungi, they display higher isotope enrichment than autotrophic plants (Post 2002, Trudell et al. 2003, Roy et al. 2009, Liebel et al. 2010). The unusually high enrichment in δ^13^C and δ^15^N is an important feature of shaded forest orchids (Gebauer and Meyer 2003, Cameron et al. 2006).

**Figure 1:**
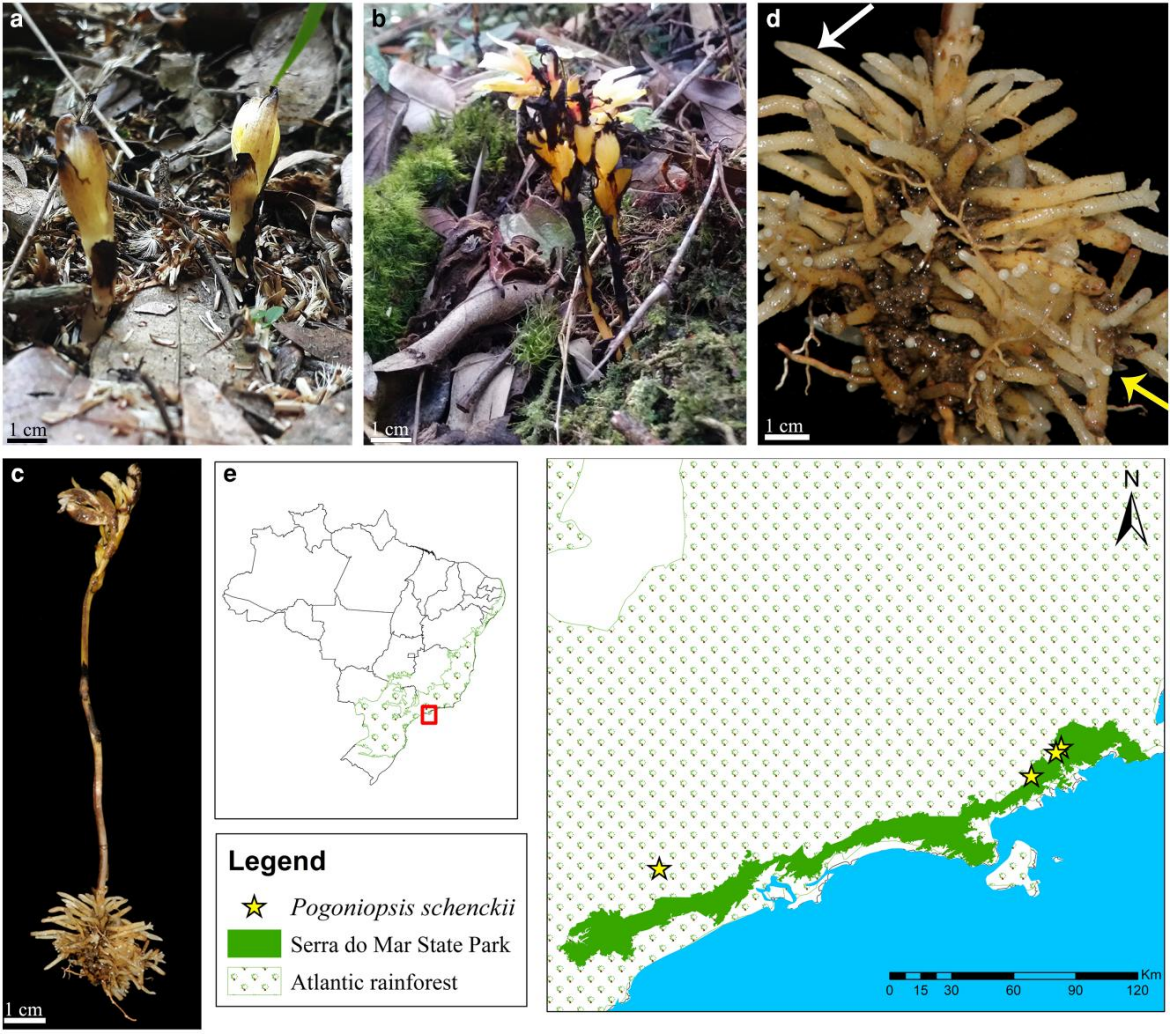
Morphology and habitat of *P. schenckii*. (a) Beginning of development out of the ground. (b) Field population with flowers and fruits. (c) Complete external morphology of the plant. (d) Details of the underground system, showing the roots with acute apex (white arrow) and rounded apex (yellow arrow). (e) Map of Serra do Mar State Park with location of collections.

Studies of mycoheterotrophic orchids from tropical regions and across different subfamilies contribute to a better understanding of the nutritional mode of these plants and highlight the role of morphological and anatomical traits in shaping the mycoheterotrophic life history. *Pogoniopsis schenckii* Cogn. is a rare tropical mycoheterotrophic orchid that is achlorophyllous, small in stature (8–15 cm), and varies with colouration from brown to yellow. It lacks leaves, has a very small stem, and its above-ground portion consists of the floral stem, which has flowers, fruits, and bracts throughout its length (Fig. 1b and c). *Pogoniopsis schenckii* is endemic to Brazil and is found only in the Cerrado savannas and Atlantic Forest (Barros et al. 2012). In a recent study, the orchid’s phylogenetic position and plastid genome evolution were evaluated, placing *P. schenkii* within the subfamily Epidendroideae, near the Sobralieae tribe (Klimpert et al. 2022). This study showed a significant genome reduction in the achlorophyllous orchid, including the loss of genes involved in photosynthesis, shedding light on the evolutionary adaptations of orchids, and offering valuable clues about the ecological niche and survival strategies of *P. schenkii* within its botanical context.

In this study, we aimed to elucidate the nutrient acquisition strategies and vegetative organ anatomy of *P. schenckii* by integrating anatomical, histochemical, and stable isotope analyses. Fully mycoheterotrophic orchids exhibit a wide range of root morphologies and fungal interaction strategies, reflecting evolutionary variation in carbon and nitrogen acquisition and in the processing of fungal pelotons formation within cortical tissues (Rasmussen 1995, Selosse et al. 2011). Such diversity suggests that root anatomical differentiation may play a key role in mediating functional specialization in mycoheterotrophic systems.

We therefore tested two overarching hypotheses. First, we tested whether if *P. schenckii* exhibits two distinct root morphotypes with contrasting functional roles: one primarily associated with carbohydrate storage, characterized by starch accumulation and limited fungal colonization, and a second morphotype densely colonized by tightly coiled pelotons typical of orchid mycorrhizas. We further propose that these morphotypes may reflect variation in peloton processing strategies, potentially representing an evolutionary transition from tolypophagy (intracellular digestion of intact pelotons) towards ptyophagy (digestion following peloton disaggregation), as described in other orchid lineages (Rasmussen 1995, Kuga et al. 2014). Second, we tested whether *P. schenckii* follows a predominantly heterotrophic nutritional strategy, acquiring most of its carbon and nitrogen via fungal intermediaries, mobilizing resources from soil- and litter-derived organic matter, rather than directly from neighbouring photosynthetic plants. In neotropical forest environments, characterized by rapid litter turnover and intense nitrogen cycling, such a strategy is expected to result in enriched stable isotope signatures (δ^13^C and δ^15^N) relative to surrounding photosynthetic vegetation and litter, resembling isotopic patterns commonly associated with decomposer-based or ectomycorrhizal nutrient pathways (Bidartondo et al. 2002, Mayor et al. 2009, Merckx and Freudenstein 2010).

Together, these hypotheses provide a mechanistic framework linking root anatomical dimorphism, fungal interaction strategies, and trophic modes in achlorophyllous orchids. By testing them in *P. schenckii*, our study contributes to a deeper understanding of mycorrhizal specialization in tropical ecosystems and highlights this species as a valuable model for investigating the evolution of plant–fungus nutritional interactions.

## Material and methods

Samples of underground organs of *P. schenckii* were collected from populations located along different trails in the Núcleo Santa Virgínia from Serra do Mar State Park, in the municipalities of São Luiz do Paraitinga (coordinates: −23.336385, −45.145806) and São Lourenço da Serra (coordinates: −23.859580, −46.939879), both in the state of São Paulo, Brazil. The distance between trails in São Luiz do Paraitinga can reach up to 40 km, separated by a highway, while the distance between the trails of the two municipalities is approximately 300 km (Fig. 1). Voucher specimens were deposited in the herbarium of the State University of Campinas (UEC) (Voucher No. 196921). Collections were conducted between December and March, during the Brazilian summer, when *P. schenckii* plants emerge above ground and populations become detectable. Outside this reproductive window, individuals remain entirely subterranean, and active searches would require extensive soil disturbance that could jeopardize population integrity and potentially threaten the persistence of this rare species. We found five distinct populations and collected 10 individuals from each for morphological and anatomical analysis and between 5 and 15 individuals for stable isotope analysis. Another limiting factor for collection is the small number of individuals within populations, along with the year-to-year fluctuations in their abundance. When the plants emerge, they produce a floral stem, with flowers that rapidly develop and self-pollinate (Alves et al. 2021). Consequently, from December to March, the fruits develop.

Metabolic profiling was not performed, as our focus was on anatomical characterization and stable isotope analyses.

### Morphological analysis

Samples of 5 mm vegetative structures of *P. schenckii* were selected. The material was fixed in Karnovsky’s solution (Karnovsky 1965) for 24 hours at room temperature. Subsequently, samples were dehydrated through a graded ethanol series, then infiltrated and polymerized with plastic resin according to the manufacturer's instructions (Leica Historesin^®^). The polymerized blocks were sectioned at 5 µm thickness using a manual rotary microtome RM 2125 RTS (Leica^®^). Sections were stained with 0.05% toluidine blue (Sakai 1973) prepared in phosphate buffer and citrate at pH 4.5, and mounted in ‘Entellan^®^’ synthetic resin (Merck^®^) (Pena-Passos et al. 2022). To document the results, images were captured using an Olympus DP71 video camera coupled to the Olympus BX 51 microscope. The anatomical studies were performed at the Laboratory of Plant Anatomy—Department of Plant Biology and at the Laboratory of Electron Microscopy—Institute of Biology, at the State University of Campinas (UNICAMP)—Brazil.

Freehand sections were prepared from fresh roots of *P. schenckii*. The histochemical tests listed in Table 1 were applied to these cuts. For the documentation of the results, the images were captured using an Olympus DP71 camera coupled to an Olympus BX 51 microscope at the laboratory of Plant Anatomy, Institute of Biology, UNICAMP.

**Table 1:** Histochemical tests applied to the roots of *P. schenckii*.

Metabolite groups		Reagent
Lipids	Total lipids	Sudan IV (Pearse 1985) Neutral red (Kirk 1970)^a^
Polysaccharide	Starch Total starch and polysaccharide	Iodinated zinc chloride (Jensen 1962) PAS ‘Periodic acid Schiff’ (McManus 1948)
	Acid mucilage Pectins	Ruthenium red (Johansen 1940) Corifosphine (Weis et al. 1988)^a^
Proteins	Total proteins	Xylidine ponceau (Vidal 1970)
Phenolic compounds	Total phenolics compounds	Ferric chloride (Pizzolato and Lillie 1973)

^a^Fluorescence induction.

For scanning electron microscopy (SEM) analysis of sample surfaces, roots of *P. schenckii* were fixed in Karnovsky’s solution (Karnovsky 1965), dehydrated through a graded ethanol series, and dried using the critical point method with CO_2_ in a Balzers CPD 030. The material was then mounted on metal clamps and covered with colloidal gold for 220 s using Bal-TEC SCD 050 (Pena-Passos et al. 2022). SEM imaging and recording were performed with a LEO VP 435 scanning electron microscope at 20 kV, at the Laboratory of Electron Microscopy at the Institute of Biology at UNICAMP.

The samples were fixed in 2.5% glutaraldehyde in 0.2 M sodium cacodylate buffer (pH 7.25) for 24 hours at 4°C, followed by post-fixation in 1% aqueous osmium tetroxide (OsO_4_) overnight. The material was then washed in distilled water and dehydrated through a graded ethanol series (10%–100%), with 1 hour for each step; the absolute ethanol step was repeated for three times. Subsequently, the samples were infiltrated and polymerized in LR White ‘hard grade’ resin (EMS^®^), according to the manufacturer's instructions. The ultrathin sections were contrasted with uranyl acetate (Watson 1958) and lead citrate (Reynolds 1963) and examined with a LEO 906 transmission electron microscope (Carl Zeiss, Germany) at the Laboratory of Electron Microscopy of Institute of Biology at UNICAMP.

### Stable isotope analysis

Between 5 and 15 *P. schenckii* samples were collected in 5 × 5 m plots at each location, previously demarcated according to population availability. Within each plot, phloem samples were collected from all *P. schenckii* individuals, from all neighbouring green plants, as well as litter and soil from 10 points at varying distances from *P. schenckii*, starting approximately 5 cm from the orchid root system, after removal of coarse debris and litter. Phloem samples were collected using a scalpel blade and placed in screw-top polypropylene bottles containing 10 ml of deionized distilled water. After 12 hours, the samples were removed from them, and the water was stored at −20°C.

For the phloem analysis, 1 ml of each sample was placed in tin capsules and oven dried at 60°C. This process was divided into five stages, with 0.2 ml dried at a time. After drying, the capsules were sealed with forceps and weighed on a precision scale. In addition to phloem, plant samples such as leaves from neighbouring green plants were collected from all individuals. These leaves were oven dried, ground, weighed in the appropriate amount for measurement in a mass spectrometer, and sealed in tin capsules.

The δ^13^C and δ^15^N composition of samples were determined by continuous flow isotope mass spectrometry (Preston and Owens 1983), on a Sercon Hydra 20-22 (Sercon, UK) stable isotope ratio mass spectrometer, coupled to a EuroEA elemental analyser (EuroVector, Italy) for online sample preparation by Dumas combustion. The δ values were calculated according to the equation:


$$\delta =[(R_{\mathrm{sample}}\text{–}R_{\mathrm{standard}})/R_{\mathrm{standard}}]*1000$$

where *R* is the ratio of the heavier isotope to the lighter. δ^15^N_Air_ values are expressed relative to atmospheric N_2_ (air), and δ^13^C_VPDB_ values are expressed relative to VPDB (Pee Dee Belemnite).

Reference materials used were USGS-25, USGS-35, BCR-657 and IAEA-CH7 (Coleman and Meier-Augenstein 2014). The laboratory standard used was Wheat Flour Standard OAS/Isotope (Elemental Microanalysis, UK). Stable isotopes analyses were performed at the Stable Isotopes and Instrumental Analysis Facility, at the Faculty of Sciences, University of Lisbon—Portugal.

The uncertainty of the isotope ratio analysis, calculated from 6 to 9 replicates of laboratory standard interspersed among samples in every batch, was ≤0.1‰. The major mass signals of N and C were used to calculate total N and C abundances, using Wheat Flour Standard OAS (Elemental Microanalysis, UK, with 1.47%N, 39.53%C) as elemental composition reference materials.

### Statistical analyses

To examine variations in each δ^13^C and δ^15^N (response variables) across sampling sites (Sao Lourenco, Poço do Filho, Piraptinga), and among types of sampled material (*P. schenckii*, photosynthetic plants, litter, soil) as fixed effects, we employed a Generalized Least Squares model (GLS). This approach accommodates correlated errors and/or unequal variances (Bai et al. 2021). Post-hoc Tukey tests were conducted to assess significant differences among the means associated with the fixed effects. Additionally, a stable isotopic mixing model was used to account for the isotopic composition of δ^13^C and δ^15^N together in the orchid *P. schenckii*, assessing its similarity or dissimilarity to the isotopic composition of potential resource sources. The ‘*simmr*’ package in R was employed for solving mixing model equations for stable isotopic data within a Bayesian framework (Parnell 2016). This mixture model identifies the probabilistic distribution that determines the origin of consumer resources in relation to potential resource pools. Monte Carlo algorithms via the Markov chain were employed to successively assume values for diet proportions until finding values that best fit the data. Initial assumptions, often poor, were discarded in the early stages, and subsequent iterations were stored for the posterior distribution—providing the best estimates of diet proportions given the data and the model. The model was fitted to generate 100 000 estimates, followed by an evaluation across different research areas. The model's outcome reveals the proportion (ranging from 0 to 1) that the consumer, *P. schenckii*, is utilizing resources from the assessed sources. In this scenario, the model incorporates two isotopic tracers (δ^13^C and δ^15^N), three sources (photosynthetic plants, litter, and soil), and one consumer, *P. schenckii*.

## Results

### Morphology and anatomical structure

**Figure 2:**
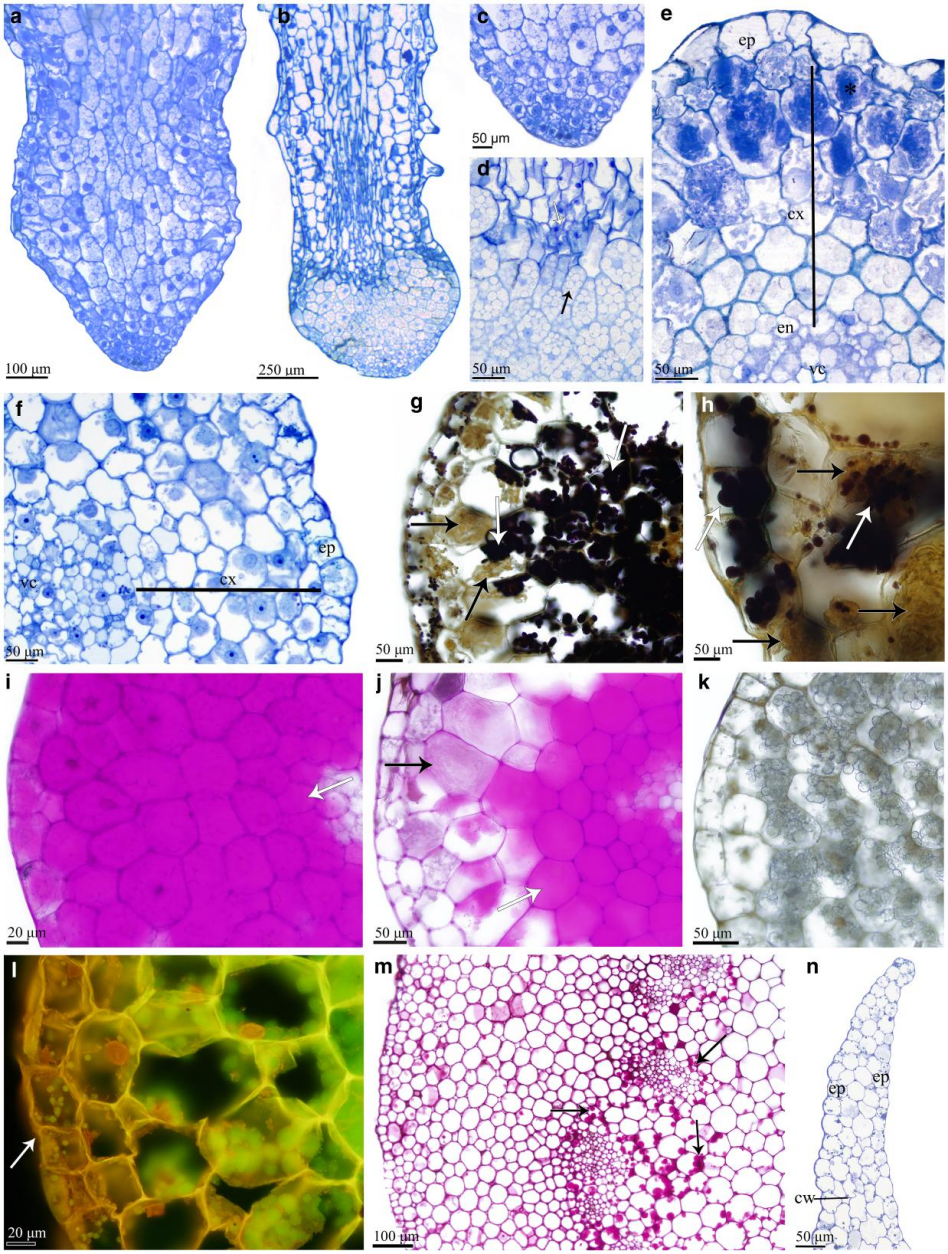
Anatomy and histochemistry of *P. schenckii*. (a) Longitudinal section of the root with acute apex. (b) Longitudinal section of the root with a rounded apex. (c) Detail of the acute apex. (d) Difference between acute apex cells with starch (black arrow) and other root cells (white arrow). (e) Root cross section with a rounded apex with hyphae in the cortex (*). (f) Root with acute apex in cross section, with the cortex cells without intercellular spaces. (g, h) Root with apex rounded with iodinated zinc chloride showing starch (white arrows) and fungal hyphae (black arrows). (i, j) Root with a rounded apex in reaction with PAS revealing starch and polysaccharides (white arrows) and hyphae (black arrow). (k) Negative reaction rounded apex root for phenolic compounds using ferric chloride. (l) Root with a rounded apex with corifosphine showing pectins in epidermal cells (arrow). (m) Cross section of the floral stem with PAS showing small starch grains around the vascular cylinder and marrow (arrows). (n) Cross section of the bracts with thin cuticle and the reduced vascular bundles (arrow). (ep = epidermis, cx = cortex, en = endoderm, vc = vascular cylinder, cw = cell wall.)

In the adult phase, *P. schenckii* has a well-developed underground system with two different root types: some with acute apex (Fig. 1d white arrow; Fig. 2a) and others with a rounded apex, displaying white to yellow colouration (Fig. 1d yellow arrow; Fig. 2b). This underground system developed in the superficial soil layer (approximately 5 cm), in association with the litter. The roots range from 3 to 10 mm in length and 1 to 2 mm in diameter. Roots with a rounded apex are generally shorter and narrower in diameter compared with roots with acute apex. Both root types are delicate and easily broken when handled. All roots had unicellular and attenuated absorbent hair (Fig. 2b).

No root cap was observed in either root type. Velamen formation was also absent in the present study. The epidermis remained unstratified along the entire root length (Fig. 2a–c). Roots with a rounded apex did not exhibit features typical of root apical meristem cells. No vestiges of the promeristem or evidence of division in apical initials were found (Fig. 2c and d). Although no physical separation was observed between the rounded apex and the rest of the root, longitudinal sections revealed a clear distinction between the cells at the apex—specialized in storing reserve substances—and cells of the vascular cylinder and cortex located farther from the apex (Fig. 2d). The endodermis contained cells without thickening, resembling those of the cortex. Casparian strips were not observed (Fig. 2e and f).

The cortex of *P. schenckii* roots contains cells with diverse functions: some are specialized in storing substances, while others cells accommodate fungal hyphae. However, certain cells simultaneously store reserves and contain hyphae within their contents (Fig. 2g and h). The cells containing hyphae were restricted to the epidermis and the outermost cortical layer (Fig. 2e, g and h). Histochemical analyses revealed marked differences in metabolite distribution between the two root morphotypes (Table 2). The semi-quantitative scale employed reflects the relative intensity and frequency of positive reactions across all sampled sections, where ‘+’ indicates a weak or sporadic reaction and ‘+++++’ denotes a strong and widespread reaction observed in most cells. Among the metabolites tested, starch exhibited the highest scores, with an intense and homogeneous accumulation in rounded-apex roots, whereas lipids, proteins, and pectins showed depletion and more localized reactions. Phenolic compounds were not detected in any of the samples.

**Table 2:** Results of histochemical tests applied to *P. schenckii* (− absence; + minimum presence; +++++ maximum presence, where ‘+’ indicates a weak or sporadic reaction and ‘+++++’ denotes a strong and widespread reaction observed in most cells).

Test	Substance	Acute apex root	Rounded apex root
Sudan IV	Lipids	+	+
Neutral red	Lipids	+	−
Iodinated zinc chloride	Starch	+++	+++++
PAS (Schiff’s reagent)	Total starch and polysaccharide	+++	+++++
Ruthenium red	Acid mucilage and polysaccharides	−	−
Corifosphine	Pectins and polysaccharides	+	+
Xylidine ponceau	Proteins	+++	++
Ferric chloride	Phenolic compounds	−	−

Starch accumulation was recurrent and present in the form of complex, numerous grains (Fig. 2g–j), including deposits in the epidermis (Fig. 2h and i). In the roots with rounded apices, all the apical cells stored starch (Fig. 2i). In cross-section of the median region, a reduction in starch grains along the organ was observed and the location of this polysaccharide was restricted to the contour of the vascular cylinder (Fig. 2j). The tests also showed that the presence of lipids and proteins in the cortex and epidermis of all the roots was insignificant. Phenolic compounds were not found (Fig. 2k), and pectins were detected inside and on the walls of the epidermis cells of both root types (Fig. 2l).

*Pogoniopsis schenckii* has extremely small subterranean stems from which adventitious roots emerge. Above the soil, conduction and support are provided by the floral stem, characterized by well-developed medulla, a small vascular cylinder, and cortical parenchymal cells with isodiametric walls (Fig. 2m). Some stems are specialized for starch storage, mainly near the vascular cylinder and medulla. This species lacks true leaves, but presents small bracts inserted in the floral stem, with a thin cuticle on the adaxial epidermis and turgid parenchymal cell in the mesophyll (Fig. 2n). The vascular cylinder is reduced to a very small central vein. The fungi were found in all vegetative structures, including in the floral stem and bracts, but in lower abundance compared with underground parts.

### Accumulation of starch and fungi in root cortex

**Figure 3:**
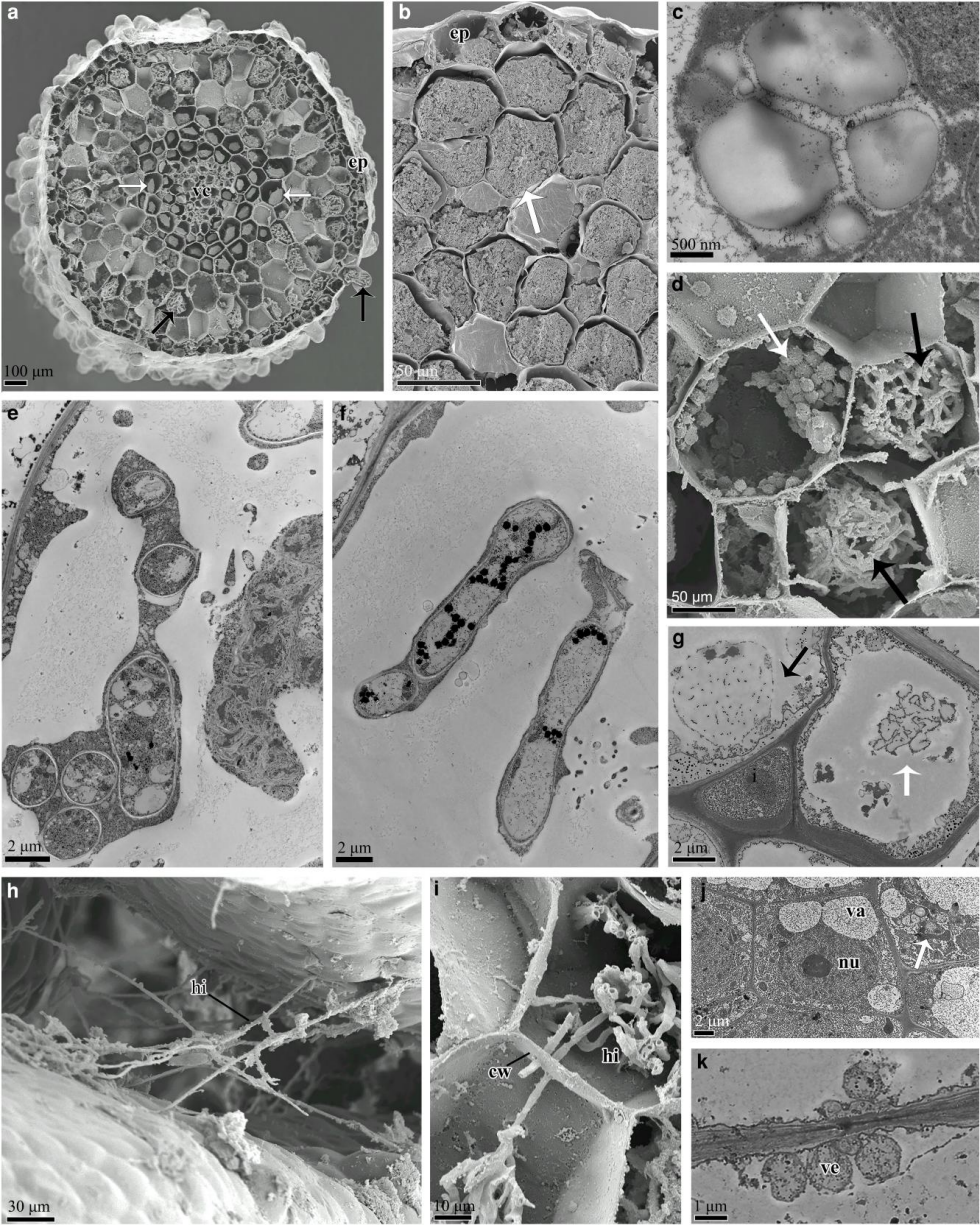
Electron microscopy of *P. schenckii*. (a) Cross section of the root with acute apex in SEM showing starch (white arrows) and hyphae (black arrows). (b) Cross section of the rounded apex root showing complex starch grains (white arrow). (c) Complex starch grain in TEM. (d) Acute apex in cross section with starch (white arrow) and hyphae (black arrows). (e) Cross sections of intact hyphae within root cells. (f) Longitudinal sections of intact and septa-free hyphae within root cells. (g) TEM of acute apex with intact hyphae (white arrow) and being digested (black arrow). (h, i) SEM of rounded apex roots showing fungi on the surface and crossing a cell wall. (j) Root cells with acute apex on TEM demonstrate living cells with high metabolic activity. (k) Floral stem in TEM evidence of very vacuolated cells with many vesicles in the cell walls. (ep = epidermis, vc = vascular cylinder, hi = hyphae, cw = cell wall, nu = nucleus, va = vacuole, ve = vesicles).

All the rounded apices (Fig. 3a) and cell layers near to the vascular cylinder (Fig. 3b) contained starch grains. These starch grains were usually large, located within complex amyloplasts, and often occupied the entire cell volume due to their abundance (Fig. 3c). Many intact hyphae were observed within cortical parenchymal cells (Fig. 3d–f). In younger regions near the apex, the hyphae appeared semi-tangled but did not form typical pelotons (Fig. 3d). Septa were not detected in the fungal hyphae; however, electro-dense material was found within the perifungal membrane surrounding the hyphae inside plant cells (Fig. 3e and f). Hyphae undergoing lysis were found farther from the apex, appearing degraded and seemingly reabsorbed by the plant cells (Fig. 3g).

We observed hyphae in the external portion of the root epidermis, forming connections between adjacent roots (Fig. 3h). These fungi were able to cross cell walls within the organ and efficiently colonize root tissue (Fig. 3i). They access the root through the epidermis, including through root hairs (Fig. 3a). The root cells of *P. schenckii* remained alive, displaying intact nuclei and many organelles, indicative of high metabolic activity, even in the presence of hyphae throughout the organs (Fig. 3j). At later development stages, farther from the apex, vacuoles expand to occupy nearly the entire volume of both root cortical cells and the floral stem cells. Many vesicles were present along the walls of all cells, and intact hyphae were no longer detectable (Fig. 3k).

### Stable isotope patterns

**Figure 4:**
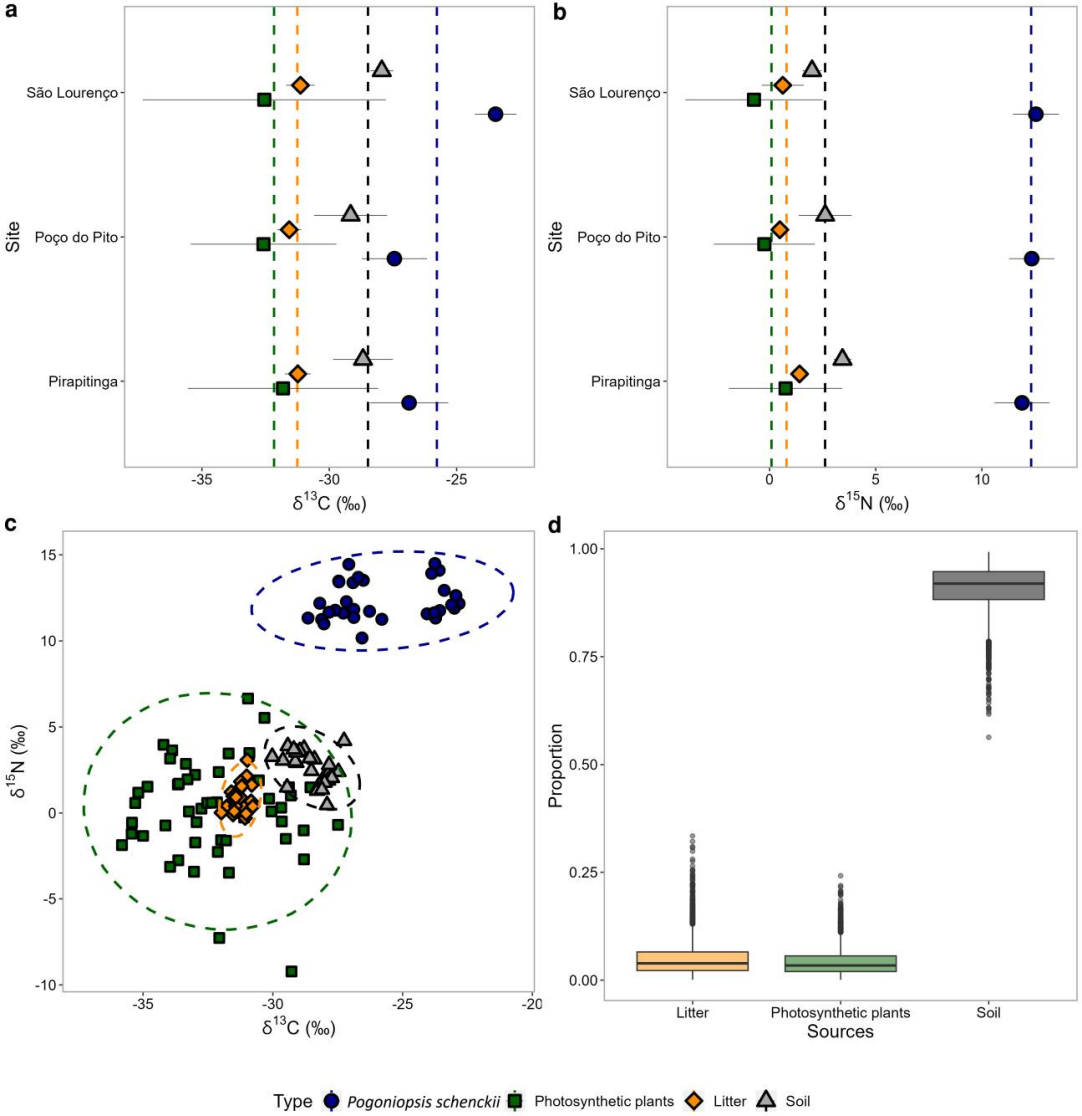
Overall results derived from stable isotopic analysis of δ^13^C and δ^15^N. (a) and (b) showcase mean values (points) and standard deviation (horizontal bar around the point) of δ^13^C and δ^15^N composition for each type of sampling collected (*P. schenckii*, Photosynthetic plant, litter, soil) across different sites (Sao Lourenco, Poco do Filho, Piraptinga). Vertical dashed lines in each panel indicate the mean value of each sampling type across all sites. (c) presents the bivariate stable isotopic space between δ^13^C and δ^15^N composition, offering a comprehensive depiction of data dispersion through data ellipses, enabling a visual assessment of central tendency and spread within each sampling type (dashed circles). (d) displays a box plot summarizing the main results of the isotopic mixing model, revealing the proportion (ranging from 0 to 1) of resources utilized by the consumer, *P. schenckii*, from the assessed sources (photosynthetic plants, litter, soil).

A notable difference exists among the sampled types—photosynthetic plants, soil, litter, and *P. schenckii*—in both δ^13^C (GLS model; *F* = 132.07; *P* < .01) and δ^15^N (GLS model; *F* = 301.09; *P* < .01) across study sites (Fig. 4a and b; mean values represented by dashed lines). We did not identify site effects for either δ^13^C or δ^15^N (GLS model; *P* > .05; Table 1). On average, the mean δ^13^C for photosynthetic plants (δ^13^C = −32.16 ± 0.18‰; ±standard error) and litter (δ^13^C = −31.25 ± 0.33‰) was similar (green and orange lines in Fig. 4a and b). The mean δ^13^C in the soil (δ^13^C = −28.48 ± 0.33‰) was significantly more enriched in δ^13^C than in photosynthetic plants and litter, and less enriched than δ^13^C in *P. schenckii* (Post Hoc Tukey test; Fig. 4a; black dashed line). *Pogoniopsis schenckii* exhibited an enrichment in δ^13^C (δ^13^C = −25.77 ± 0.33‰) than any other evaluated group (Post Hoc Tukey test; Fig. 4a, blue dashed line).

A similar pattern was observed for δ^15^N. Photosynthetic plants (δ^15^N = 0.09 ± 0.25‰) and litter (δ^15^N = 0.79 ± 0.39‰) showed similar mean values (Post Hoc Tukey test; *P* > 0.05; Fig. 4b green and orange dashed lines). The soil δ^15^N was significantly more enriched (δ^15^N = 2.61 ± 0.25‰) (Post Hoc Tukey test; *P* < 0.05; Fig. 4b, black dashed line). On the other hand, *P. schenckii* exhibited a δ^15^N more enriched than any other factor evaluated (δ^15^N = 12.32 ± 0.33‰) (Post Hoc Tukey test; Fig. 4b, blue dashed line). The enrichment was 4.7 times greater than that of the soil, and 28 times greater than the combined mean of photosynthetic plants and litter. In summary, the stable isotopic signatures of δ^13^C and δ^15^N in *P. schenckii* were distinctly different from those of the other sources evaluated (Fig. 4c). The observed similarity is primarily attributed to some overlap between the δ^13^C composition of the soil samples and *P. schenckii* samples.

The isotopic mixing model showed low explanatory power, with only 30% agreement between observations and predictions of the evaluated tracers (*simmr* model output not shown). Although the estimated contribution proportions remain somewhat uncertain, the dominant trend of soil as the main nutrient source was captured. Model outputs consistently indicate that the soil is the most likely primary resource source for *P. schenckii*, with photosynthetic hosts contributing only minimally (Fig. 4d). When examined separately by area the results continued to point to the soil as the dominant contributor to *P. schenckii’*s resource acquisition (*simmr* model output not shown).

## Discussion

Confirming one of our initial hypotheses, the root system of *P. schenckii* exhibits two distinct morphologies: (i) a morphotype with rounded apex and a substantial starch reserve replacing its entire meristematic portion, and (ii) a morphotype with an acuminate apex and a reduced meristematic portion. Unexpectedly, fungal hyphae were observed growing around and colonizing the roots, forming semi-coiled pelotons within root cells. These structures differ from the densely coiled pelotons commonly reported for the family (Huynh 2004, Zhu et al. 2009, Sathiyadash et al. 2012). Furthermore, stable isotope analysis indicates that soil-derived resources are the primary source of nutrients, with nutrients transferred to the orchid through fungal partners. Supporting our hypothesis, *P. schenckii* shows a relative enrichment in δ^13^C and, particularly, in δ^15^N compared to any other evaluated source, suggesting transfer via ectomycorrhizal fungi associated with photosynthetic plant species.

### Morphology and anatomical structure

The root system of *P. schenckii* exhibits two root morphotypes, a feature commonly observed in mycoheterotrophic species (Sainge et al. 2005, Martos et al. 2009, Imhof et al. 2013). For instance, the achlorophyllous orchid *Wullschlaegelia aphylla* (Sw.) Rchb. f. also presents two root morphotypes: one comprising thinner, elongated roots, and the other comprising thicker and shorter tuberous roots (Martos et al. 2009). Additionally, the filiform roots of these species have been reported to be interconnected by hyphae or attached to decomposing wood or leaf litter (Sainge et al. 2005, Martos et al. 2009, Imhof et al. 2013).

The two root morphotypes of *P. schenckii* also exhibit distinct patterns of length and thickness. The rounded-apex roots were thinner and shorter than the acute-apex roots, which are comparatively longer and thicker. Interestingly, this characteristic opposes a phenomenon frequently observed in mycoheterotrophic species, known as the ‘Mycoheterotrophy Dilemma’ where there is a predominant occurrence of either short and thick roots or thin and long roots in these species (Imhof 2010). The root dimorphism pattern exhibited by *P. schenckii* may reflect its low mycorrhizal specificity combined with its seed dispersal strategy. The species produces mature, indehiscent fruits that fall immediately beneath the parent plant (Alves et al. 2021), whose roots already host established mycorrhizal partners. We suggest that rounded-apex roots could play a primary role in soil exploration and recruitment of mycorrhizal fungi. However, because of their immediate access to the parent plant's established mycorrhizae, this root morphotype appears to have lost that function. Therefore, we suggest a unique model of root dimorphism in *P. schenckii*, in which the rounded-apex roots gradually lose their ability to elongate throughout evolution, prematurely converting their meristematic apex into an energy storage region. This hypothesis is also supported by unpublished work by Sisti (2024), which identified various cosmopolitan fungi as mycorrhizal partners of *P. schenckii*, reinforcing the species’ ease in recruiting its fungal partners.

Root dimorphism is often related to different functions performed by each morphotype (Moreira and Isaias 2008). Given the presence of substantial starch reserves and peloton colonization in both root morphotypes of *P. schenckii*, our results suggest functional differentiation between them. Roots with an acute apex appear to play a more prominent role in mineral nutrient uptake and substrate anchorage, consistent with the functional specialization of distal, actively growing root portions in resource acquisition and soil interaction (Eissenstat and Achor 1999, McCormack et al. 2015). In contrast, roots with a rounded apex may function primarily as storage and maintenance structures, contributing to the accumulation of carbon reserves and other metabolites, as well as to the preservation of fungal symbionts. Their smaller size and closer proximity to the stem may reduce mechanical damage and facilitate rapid resource transport and remobilization. This interpretation is supported by the dynamic role of pelotons in nutrient exchange and intracellular cycling within orchid roots (Rasmussen 2002, Dearnaley et al. 2012).

*Pogoniopsis schenckii* may be undergoing an evolutionary process in which one of its root morphotypes is being lost, retaining only the acute-apex roots that dominate its root system. There is a clear evolutionary trend in mycoheterotrophic species towards the development of stronger roots with an enlarged cortex capable of accommodating mycorrhizae while storing carbohydrates and other compounds. Without the need for extensive soil exploration to find their mycorrhizal partners, the acute-apex roots could meet the plant's need for storage and carbon acquisition through fungi. Species with a single root morphotype, being short and thick, can be found in orchids such as *Neottia nidus-avis* (L.) Rich. and *Wullschlaegelia calcarata* Benth, being even more common than dimorphism (Imhof et al. 2013). According to Imhof (2010), a more voluminous cellular cortex, as in the case of thicker primary roots, is especially advantageous as it provides more space for mycorrhizal colonization, allowing for a greater fungal content to be digested.

The dependence on mycorrhizal associations due to a mycoheterotrophic lifestyle has profoundly impacted the underground architecture of *P. schenckii*, which can be considered essentially an underground orchid since the species does not need to maintain an above-ground photosynthetic system. The vegetative system composed of underground stem and roots is the only perennial part of the species, while the reproductive organs are visible above ground only during its reproductive phase, which occurs between December and March during the Brazilian summer. Other adaptations such as reduced stem and root size may represent resource economy that will be used during its reproductive phase. We also believe that the loss of promeristem activity in both root morphotypes occurs during the development of the above-ground part, potentially being active only during the vegetative phase of *P. schenckii*. This dependence on fungal association is also related to attenuated root hairs, which are common in achlorophyllous plants such as *Voyria aphylla*, *V. truncata*, and *V. tenella* from the Family Gentianaceae to function as a doorway to mycorrhizal fungi in the plant cells (Row and Reeder 1957, Baylis 1972, 1975, Imhof 1997, 1999, Imhof and Weber 1997).

### Accumulation of starch and fungi in root cortex

All the starch found in the cells of *P. schenckii* is stored in amyloplasts, an important carbon reserve essential for the survival of this achlorophyllous orchid, since the species lacks chloroplasts. The lack of photosynthesizing tissue causes an extreme condition in which a high level of long-term starch storage can be required in these individuals. In this context, the starch produced in root tissues functions as a carbon reserve, which can be accessed under adverse environmental conditions or during a specific phase of the plant's life cycle (Tetlow et al. 2004). Besides being vital in various processes, starch reserves can also be remobilized to supply high carbon demands, such as the production of flowers and fruits during the reproductive cycle of *P. schenckii*.

We suggest that the complex architecture of the starch grains synthesized by *P. schenckii* could represent a strategy of the plant to prevent or, at least, discourage their utilization by the associated fungi. The architectonically complex starch grains found abundantly in the roots of *P. schenkii* are insoluble glucose polymers and the complexity of this type demonstrates that the structure involves two types of chains: amylose and glucans (Tetlow et al. 2004). The synthesis of complex grains such as in *P. schenckii* is only possible by the interaction of biosynthetic enzymes that may also be involved in the degradation of these grains (Tetlow et al. 2004), and such enzymes may be absent in their fungal partners.

The pattern of colonization and degradation of pelotons in *P. schenckii* can be characterized as a process known as tolypophagic. In this process, pelotons are formed and completely degenerated (Burgeff 1932). During tolypophagic, a division may occur between outer cortical cell layers reserved for the colonization of hyphal coils that will not be digested and inner layers where digestion occurs (Burgeff 1932). Generally, such division can be observed in *P. schenckii* roots, along with a second pattern found where pelotons are preferentially degraded by the plant in a longitudinal direction, starting from the base towards the apex of the roots, with this process intensifying during the reproductive phase. According to Imhof (2010), patterns of mycorrhizal colonization, such as those that distinguish between areas where hyphae are kept alive and where they are digested, allow for sustainable use of the fungus and longevity of the association.

The semi-coiled architecture of pelotons found in *P. schenckii* may represent the beginning of the species’ transition to a ptyophagic *process*. In this process, only the tips of the hyphae penetrate the plant's digestion layers, releasing their contents without coiling (Burgeff 1932, Campbell 1962, 1963, 1964, Rasmussen and Rasmussen 2014, Li et al. 2020). There is speculation that this process could be interpreted as an evolutionary progression of orchid mycorrhizae, from an undifferentiated colonization pattern to tolypophagic, and finally to ptyophagic (Peterson et al. 2004, Imhof et al. 2013). The presence of electron-dense content found inside the peri-fungal membrane in the root cells of *P. schenckii* may indicate active transfer of compounds from the fungus to the plant without hyphal degeneration, which is essential in this transition.

No fungi with septate hyphae were found in the organs of *P. schenckii*, which represents one of the diagnostic characteristics of Basidiomycota, although a basidiomycete fungus of the genus *Tulasnella* was identified inhabiting the roots of *P. schenckii* in a previous study (Sisti et al. 2019). It was believed that the challenge of isolating and finding basidiomycete fungi in the organs of *P. schenckii* was due to their lower abundance compared to ascomycetes (Sisti et al. 2019, Alves et al. 2021). However, a recent study demonstrated that the ratio of fungi from the phyla Basidiomycota and Ascomycota is comparable in different organs, with basidiomycetes being even more predominant in root and floral stalk (Sisti 2024). Indeed, the septation of fungal hyphae is difficult to visualize under light microscopy, especially when coiled inside plant cells, and even in transmission electron microscopy, the visualization of small sections of hyphae does not always allow for this determination.

The fungi that penetrate the cells of two nearby roots of *P. schenckii* forming a type of hyphae bridge, may have an important role in integrating the underground system of this orchid. In his investigations, Imhof (2010) reports the discovery of eleven colonization patterns in thirteen mycoheterotrophic species, in addition to an additional pattern in orchids, involving not only roots but also rhizomes, stems, and even leaves. The various patterns formed enable different functions within the plant, such as long-distance and short-distance distribution, reserve storage, and digestion of fungal hyphae (Imhof 2010). In *P. schenckii*, at least three patterns were observed, with semi-coiled pelotons being kept intact in the outer layers of the epidermis and cortex of both types of roots, degraded pelotons being digested in the inner layers, and straight hyphae emerging from within one root to enter other roots of the plant, connecting the orchid's root system. This latter pattern likely connects the roots of other individuals of the species, or even an entire population, allowing direct communication between the hyphal complexes inhabiting them and their underground systems.

### Accessing *P. schenckii* nutrition system through stable isotopes

*Pogoniopsis schenckii* was more enriched in δ^13^C compared to photosynthetic plants and was significantly more enriched in ^15^N than any component across all sampled populations. These isotopic values identify fungi as the most likely nutrient source for this species (Hobbie and Högberg 2012). This pattern remained consistent across all study sites (Fig. 4). The enrichment in both δ^13^C and δ^15^N observed in *P. schenckii* is consistent with isotopic signatures reported for other mycoheterotrophic plants (Merckx and Freudenstein 2010, Courty et al. 2011, Hynson et al. 2016, Gomes et al. 2020). Mycoheterotrophic species associated with ectomycorrhizal or saprophytic fungi are typically more enriched in δ^13^C and δ^15^N than autotrophic plants (Suetsugu et al. 2020). Similar patterns have also been reported for *Wullschlaegelia aphylla* (rainforest—island of Guadeloupe), *Gastrodia similis* Bosser (rainforest—La Réunion island), and *Epipogium aphyllum* Swartz (boreal forest—Norway) (Martos et al. 2009, Liebel and Gebauer 2011).

We suggest that the isotopic signature of *P. schenckii* reflects that carbon and nitrogen are acquired through its symbiotic fungi from the soil-derived sources, as evidenced by the similarity between the isotopic signatures of the plant and that of surrounding soil. According to Ogura-Tsujita et al. (2018), the signature of mycoheterotrophic plants is enriched relative to their ultimate source but closer to their immediate source. Consequently, those associated with ectomycorrhizal fungi show highly enriched values compared to neighbouring autotrophs, yet remain isotopically similar to their associated fungi (Gebauer and Meyer 2003, Trudell et al. 2003, Bidartondo et al. 2004, Hobbie and Högberg 2012). Although our isotopic mixing model showed limited explanatory power (discussed below), it consistently identified soil as the most plausible primary source of nutrients for *P. schenckii*. Furthermore, our data also suggest that mycorrhizal partners of *P. schenckii* are not saprotrophic decomposers of litter. Mycoheterotrophic plants tend to exhibit δ^15^N enrichment (averaging ∼5‰; Mayor et al. 2009) and lower similarity to autotrophic plants (Suetsugu et al. 2020). The isotopic signatures observed in *P. schenckii* align with the former pattern; while saprotrophic fungi generally exhibit lower δ^15^N values—ranging from −4 to 4‰ (Mayor et al. 2009, Suetsugu et al. 2020)—our samples showed a markedly higher mean of 12.32 ± 0.33‰. This value falls within the established range for ectomycorrhizal fungi δ^15^N composition (−2 to 14‰; Mayor et al. 2009, Suetsugu et al. 2020). Consequently, this high ^15^N enrichment, coupled with the lack of extreme ^13^C enrichment, supports the indication of an ectomycorrhizal-mediated nutrient acquisition strategy.

The unexplained variance in the isotopic mixing model in our study may be attributed to three main factors. First, the potential influence of omitted fungal sources must be considered; since specific mycelial networks were not directly sampled, the model may not account for the full complexity of the symbiotic network or possible mycorrhizal shifts within Ascomycota groups potentially delivering different nutrient sources during plant development (Sisti et al. 2019, Li et al. 2021). In the Atlantic Forest, high litter heterogeneity and diverse fungal lineages can introduce nitrogen (N) and carbon (C) sources from multiple soil layers that are not captured by our soil or litter categories. Consequently, the isotopic signatures of these sources may reflect a mixture of elements from varying soil depths or shifted mycorrhizal associations. Second, isotopic discrimination factors (Δ) used in mixing models are well-established for animal models but remain uncertain for symbiotic fungi (Phillips and Gregg 2001). This uncertainty arises because fungi fractionate nutrients while decomposing organic matter or receiving photosynthates from ectomycorrhizal hosts. Furthermore, *P. schenckii* may possess unique metabolic pathways that alter nutrient fractionation, as the movement of nutrients from fungal hyphae to orchid cells involves additional fractionation influenced by the specific compounds transferred (e.g. amino acids vs. simple sugars). Once inside the orchid, these nutrients undergo metabolic transformations—such as starch synthesis in the roots—that further alter the isotopic balance (Hobbie and Högberg 2012). Third, mixing models are highly sensitive to source variability. A fundamental assumption of these models is that all significant sources have been identified and sampled (Phillips and Gregg 2001, Vane et al. 2023). As shown in Fig. 4c, the isotopic signatures of plants and litter exhibit significant overlap, while soil presents a small enrichment in δ^15^N and δ^13^C. Such inherent overlap in dense forest understories drastically reduces the model's mathematical resolution and predictive power (Phillips and Gregg 2001). Therefore, further studies are needed to elucidate potential mycorrhizal shifts and specific discrimination factors, as well as to better identify resource sources, to improve our understanding of mycoheterotrophic plants in the tropics.

## Conclusion

The findings of this study highlight the singularity of *P. schenckii* among tropical mycoheterotrophic orchids, revealing distinctive anatomical traits and nutritional strategies that may represent transitional stages in its evolutionary trajectory. The coexistence of two root morphotypes, combined with the presence of living hyphae and distinct peloton degradation patterns, suggests a dynamic model of fungal interaction, potentially ensuring flexible access to resources under heterogeneous environmental conditions. The strongly enriched δ^13^C and, particularly, δ^15^N isotopic signatures confirm the species’ dependence on ectomycorrhizal fungi as the primary mediators of plant carbon and nitrogen uptake, underscoring its position at higher trophic levels.

These findings expand our understanding of the spectrum of mycoheterotrophic strategies in tropical regions and suggest that *P. schenckii* may represent a model species for investigating evolutionary processes of mycorrhizal specialization and plasticity. Future studies focusing on the diversity and functional roles of its fungal partners, and deepening comparative analyses across populations, will be crucial to clarify whether these characteristics reflect an adaptive trend or a phylogenetically conserved trait.

## Supplementary Material

plag027_Supplementary_Data

## Data Availability

The data that underpin the statistical analysis in this article are shared in the Supplementary material.
